# *Stat1* is an inducible transcriptional repressor of neural stem cells self-renewal program during neuroinflammation

**DOI:** 10.3389/fncel.2023.1156802

**Published:** 2023-08-16

**Authors:** Jaime Imitola, Ethan W. Hollingsworth, Fumihiro Watanabe, Marta Olah, Wassim Elyaman, Sarah Starossom, Pia Kivisäkk, Samia J. Khoury

**Affiliations:** ^1^Laboratory for Neural Stem Cells and Functional Neurogenetics, Division of Multiple Sclerosis and Neuroimmunology, University of Connecticut Health Center, Farmington, CT, United States; ^2^Center for Neurologic Diseases, Department of Neurology, Brigham and Women’s Hospital, Harvard Medical School, Boston, MA, United States; ^3^Medical Scientist Training Program, University of California, Irvine, Irvine, CA, United States; ^4^Department of Neurology, Columbia University Medical Center, New York City, NY, United States; ^5^Institute for Medical Immunology, Charité–Universitätsmedizin Berlin, Berlin, Germany; ^6^Alzheimer’s Clinical and Translational Research Center, Department of Neurology, Massachusetts General Hospital, Harvard Medical School, Boston, MA, United States; ^7^Abu Haidar Neuroscience Institute, American University of Beirut Medical Center, Beirut, Lebanon

**Keywords:** multiple sclerosis, experimental autoimmune encephalomyelitis, neural stem cells, subventricular zone, STAT1, interferons, Sox9

## Abstract

A central issue in regenerative medicine is understanding the mechanisms that regulate the self-renewal of endogenous stem cells in response to injury and disease. Interferons increase hematopoietic stem cells during infection by activating STAT1, but the mechanisms by which STAT1 regulates intrinsic programs in neural stem cells (NSCs) during neuroinflammation is less known. Here we explored the role of STAT1 on NSC self-renewal. We show that overexpressing *Stat1* in NSCs derived from the subventricular zone (SVZ) decreases NSC self-renewal capacity while *Stat1* deletion increases NSC self-renewal, neurogenesis, and oligodendrogenesis in isolated NSCs. Importantly, we find upregulation of STAT1 in NSCs in a mouse model of multiple sclerosis (MS) and an increase in pathological T cells expressing IFN-γ rather than interleukin 17 (IL-17) in the cerebrospinal fluid of affected mice. We find IFN-γ is superior to IL-17 in reducing proliferation and precipitating an abnormal NSC phenotype featuring increased STAT1 phosphorylation and *Stat1* and *p16*^ink4a^ gene expression. Notably, *Stat1*^–/–^ NSCs were resistant to the effect of IFN-γ. Lastly, we identified a *Stat1*-dependent gene expression profile associated with an increase in the *Sox9* transcription factor, a regulator of self-renewal. *Stat1* binds and transcriptionally represses *Sox9* in a transcriptional luciferase assay. We conclude that *Stat1* serves as an inducible checkpoint for NSC self-renewal that is upregulated during chronic brain inflammation leading to decreased self-renewal. As such, *Stat1* may be a potential target to modulate for next generation therapies to prevent progression and loss of repair function in NSCs/neural progenitors in MS.

## Introduction

Transplantation of exogenous neural stem cells (NSCs) holds tremendous clinical potential evidenced by their success in ameliorating neurological diseases (Pluchino et al., 2003, 2005; Merzaban et al., 2015) in animals models and in humans in recent phase I trials (Pluchino et al., 2003; Warre-Cornish et al., 2020). In contrast, the repair potential of endogenous NSCs to injury and disease remains less explored. Initially, the self-renewal of endogenous NSCs (Molofsky et al., 2003) is increased by central nervous system (CNS) injury or acute inflammation, as seen early in multiple sclerosis (MS). However, aging and chronic neuroinflammation decrease NSC renewal and differentiation capacity over time (Imitola et al., 2003; Aharoni et al., 2005), thereby limiting the endogenous capacity of the brain for self-repair in MS. Such a pattern suggests a switch in the permissibility of the immune microenvironment from favorable to unfavorable to NSC self-renewal and differentiation, (Nait-Oumesmar et al., 2007; Pluchino et al., 2008; Wang et al., 2008; Rasmussen et al., 2011; Tepavčević et al., 2011). What exactly in the immune microenvironment or NSC response program triggers this switch, however, remains largely unknown.

One hypothesis for the lack of repair in chronic neuroinflammation is that NSCs and other progenitors like oligodendrocyte progenitor cells (OPCs) may participate in the immune microenvironment in MS by becoming activated direct targets of the immune response as they respond to inflammation by expressing immune-related molecules. The targeting may involve not only cell death but also cells intrinsic repair capacity dysfunction due to changes in the NSCs transcriptional programs (Imitola et al., 2004a; Kirby et al., 2019). Interferons have been shown to activate hematopoietic stem cell (HSC) self-renewal by inducing proliferation in response to infections (Essers et al., 2009; Baldridge et al., 2010); however, their role in the brain is still not clear. In particular, the IFN-γ/signal transducer and activator of transcription 1 (STAT1) pathway is an important participant in neuroinflammation. Recent work has found infiltrating T cells expressing IFN-γ in the aging subventricular zone (SVZ) NSC niche (Dulken et al., 2019), suggesting its effect in regulating NSC self-renewal. Indeed, IFN-γ regulates progenitor proliferation *in vivo* (Arien-Zakay et al., 2009), and activates *Stat1* and *p21* (Mäkelä et al., 2010) in NSCs. Furthermore, IFN-γ, through activation of its canonical STAT1 pathway, acts specifically on Nestin + progenitors to decrease progenitor proliferation and the number of cycling cells in a normal SVZ (Pereira et al., 2015). Moreover, a recent paper demonstrated type I interferon induces cell cycle arrest and quiescence of SVZ NSCs (Carvajal Ibañez et al., 2023). Still, the transcriptional targets of *Stat1* and the direct role of *Stat1* in the regenerative program of NSCs in neuroinflammation as seen in MS due to IFN-γ are still unknown.

Here, we investigate the role of *Stat1* in the molecular program of NSCs and demonstrate *Stat1* negatively controls self-renewal capacity in NSCs in a bidirectional manner during homeostasis and in response to inflammation. At a mechanistic level, we identify genes directly regulated by *Stat1* in NSCs, including *Sox9*, through which *Stat1* binds and transcriptionally represses this pro-neural stem cell gene (Scott et al., 2010). Our data suggest the inflammatory factors regulating self-renewal are stem cell-specific and are relevant to the search for novel therapies tailored to improve regeneration after chronic and progressive injury like MS.

## Results

### Expression of interferon-γ/Stat1 associated genes in SVZ NSCs

**FIGURE 1:**
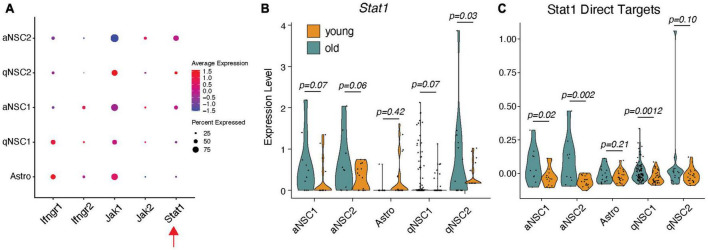
IFN-γR-Stat1 pathway activity in young and old mouse SVZ-NSCs. **(A)** Dot plot for gene expression of *Stat1* pathway members by cell type using scRNA-seq data. **(B)** Cell-type-specific violin plots for *Stat1* in different NSCs and **(C)** its direct transcriptional targets split by age. One-tailed *t*-tests. aNSC, activated NSC; Astro, astroglia; qNSC, quiescent NSCs.

Because IFN-γ-expressing T cells infiltrate the aging SVZ, we hypothesized that this might trigger simultaneous increase in *Stat1* expression in NSCs. To examine this possibility, we leveraged publicly available Smart-Seq2 single cell RNA-seq (scRNA-seq) data from young and old murine SVZ (Kalamakis et al., 2019); this dataset captures significantly more transcripts than any other scRNAseq datasets. We first examined whether the IFN-γ pathway is expressed in the SVZ. Except for minimal levels of *Jak2*, we find SVZ cell types express all members of the IFN-γ pathway, like SVZ astrocytes that are considered stem cells in the normal SVZ (Figure 1A). We next asked if *Stat1* is increased in these cell types with age. *Stat1* levels trend strongly toward increasing with age across all NSC populations, statistically significant on a subtype of quiescent NSCs (Figure 1B). Since *Stat1* is a transcription factor, we next assessed expression of its direct target genes in the SVZ of young and old mice (promoter bound; FC > 2 with IFN-γ treatment) as defined by past ChIP-sequencing experiments (Dulken et al., 2019; Figure 1C). We found an upregulation of *Stat1* target genes in older aNSCs and qNSC1, supporting the idea that inflammation associated with aging activate *Stat1* and its downstream networks in the SVZ.

### *Stat1* bidirectionally regulates NSC self-renewal

**FIGURE 2:**
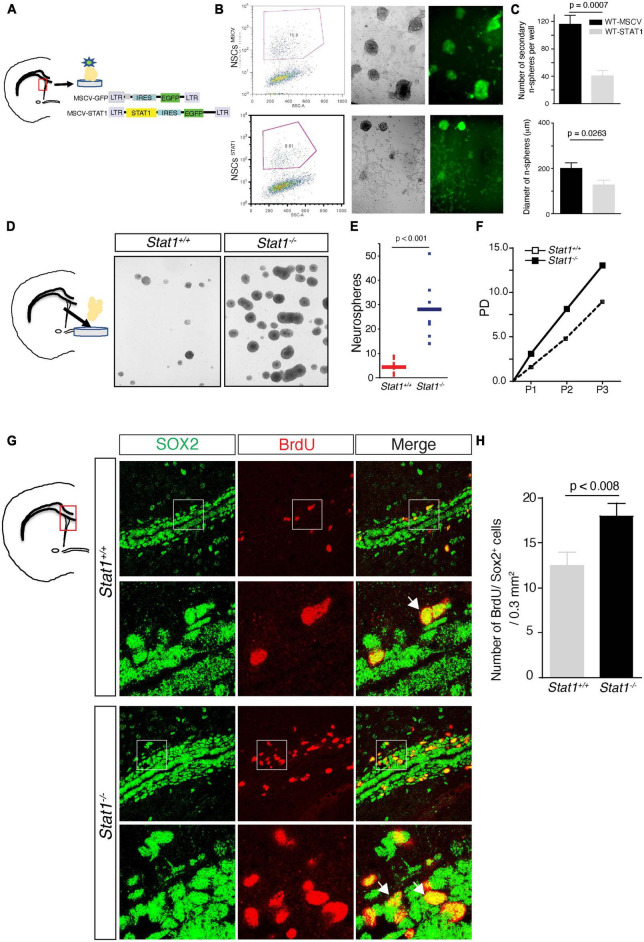
Self-renewal of NSCs after *Stat1* overexpression or ablation. **(A)** Diagram showing the construction of retrovirus delivering *Stat1* to NSCs and the isolation of SVZ NSCs **(B)** Secondary neurospheres infected with empty vector-GFP (MSCV) and *Stat1-GFP* vector (STAT1). NSCs were sorted and plated in self-renewing conditions, note the presence of GFP positive neurospheres in both conditions but diminished in GFP-STAT1 containing vector **(C)** quantification of secondary neurospheres numbers and diameter overexpressing STAT1, showing reduction of numbers and diameter compared to control *n* = *3 p* < 0.0007 by unpaired *t-test*. Data is representative of two independent experiments. **(D)** Culture of secondary neurospheres from *Stat1*^+/+^ and *Stat1*^–/–^ mice. **(E)** Quantification of number of neurospheres obtained from NSCs from *Stat1*^–/–^ and *Stat1*^+/+^ mice there is a significant increase in the numbers of neurospheres, *p* < 0.001 by *t-test*. Data is representative of three independent experiments. **(F)** Quantification of population doublings, a measure of increased lifespan on NSCs from *Stat1*^+/+^ and *Stat1*^–/–^ mice. **(G)** Representative figure of Incorporation of BrdU by Sox2 + neural progenitors in the SVZ of *Stat1*^–/–^ and control adult brain by stereological methods and confocal microscopy (arrows). **(H)** Quantification of Sox2 + BrDU + neural progenitors *in vivo* in *Stat1*^+/+^ and *Stat1*^–/–^ mice SVZ (*n* = *3) p* < 0.008 by *t-test.* Bar: 50 μm.

In a chronic model of MS, there is a decrease in NSC self-renewal in the SVZ (Pluchino et al., 2008) that is spatiotemporally correlated with increase in *Stat1*. To determine whether STAT1 plays a causative role in regulating self-renewal in NSCs, we delivered STAT1 to wild type NSCs isolated from the SVZ using a bicistronic retroviral vector LTR-STAT1-IRES-GFP containing the coding sequence of *Stat1* and *GFP* (Lesinski et al., 2003). These two genes are simultaneously expressed in infected cells and a high expression of both genes is indicated by high GFP fluorescence. These NSCs were then FAC-sorted for GFP-positive single cells to ensure retroviral transduction, gene expression, and survival and plated at clonal dilution that prevent fusion of cells to accurately measure the formation of secondary neurospheres (Molofsky et al., 2003; Pluchino et al., 2008; Rasmussen et al., 2011). At clonal dilution, the resulting secondary GFP + neurosphere numbers were then counted after 7 days *in vitro* as a measure of self-renewal capacity (Molofsky et al., 2003; Pluchino et al., 2008; Wang et al., 2008; Rasmussen et al., 2011). Overexpression of STAT1 decreased neurosphere diameter and numbers compared to the control vector-infected NSCs (*p* < 0.0001; *n* = 3 from 2 independent experiments) (Figures 2A–C). In addition, there is a small increase in cell death in the STAT1-GFP NSCs compared to control (Supplementary Figure 1A) indicating a predominant effect in proliferation as the primary mechanism rather than cell death (Pereira et al., 2015).

We next asked whether depleting *Stat1* from NSCs would have the opposite effect and increase NSC self-renewal. To test this, we measured the basal self-renewing capacity of adult NSCs isolated from the subventricular zone from *Stat1*^–/–^ mice confirmed by the absence of *Stat1* at the DNA and mRNA level in the isolated neurospheres (Supplementary Figures 1B, C). Under these conditions, using established methods (Molofsky et al., 2003; Pluchino et al., 2008; Wang et al., 2008; Rasmussen et al., 2011) revealed an increase in the number of secondary neurospheres (*p* < 0.001) and in *Stat1*^–/–^ NSC population doubling (Figures 2D–F). To demonstrate the specificity of this effect, we delivered the same retroviral vector LTR-STAT1-IRES-GFP to *Stat1*^–/–^ NSCs, sorted them at clonal dilution, and counted them 7 days later. *Stat1* overexpression rescued the reduction in NSC self-renewal caused by *Stat1*^–/–^ knockout (*p* < 0.001) (Supplementary Figures 1D–F).

Having established its role *in vitro* by isolation of SVZ NSCs, we then asked if *Stat1* regulates NSC proliferation *in vivo*. To do this, we performed BrdU injections in adult *Stat1*^–/–^ mice (that are viable and normal in appearance) and controls. BrdU was injected for 7 days and then the mice were sacrificed. Of note, these concentrations of BrDU does not change the *in vivo* outcomes and is widely used as reported by many groups (Pluchino et al., 2008; Li et al., 2010; Rasmussen et al., 2011; Starossom et al., 2019). We used Sox2, an established marker mostly expressed in NSCs and minimally in transient neural progenitors. We quantified the number of Sox2/BrdU-positive NSCs in the normal adult SVZ and rostral migratory stream (RMS). We found that *Stat1* depletion increases the number of proliferating BrdU/Sox2 + NSCs in the SVZ (*p* < 0.008 *n* = 3 mice per group), confirming the *in vitro* observations (Figures 2G, H). These data demonstrate a bidirectional role of *Stat1* in controlling NSC self-renewal, meaning increases of *Stat1* leads to decreased NSCs self-renewal and decreases of *Stat1* leads to increase in self-renewal *in vitro* and proliferation *in vivo*.

### *Stat1* knockout increases sensitivity to niche signals, self-renewal, and differentiation

**FIGURE 3:**
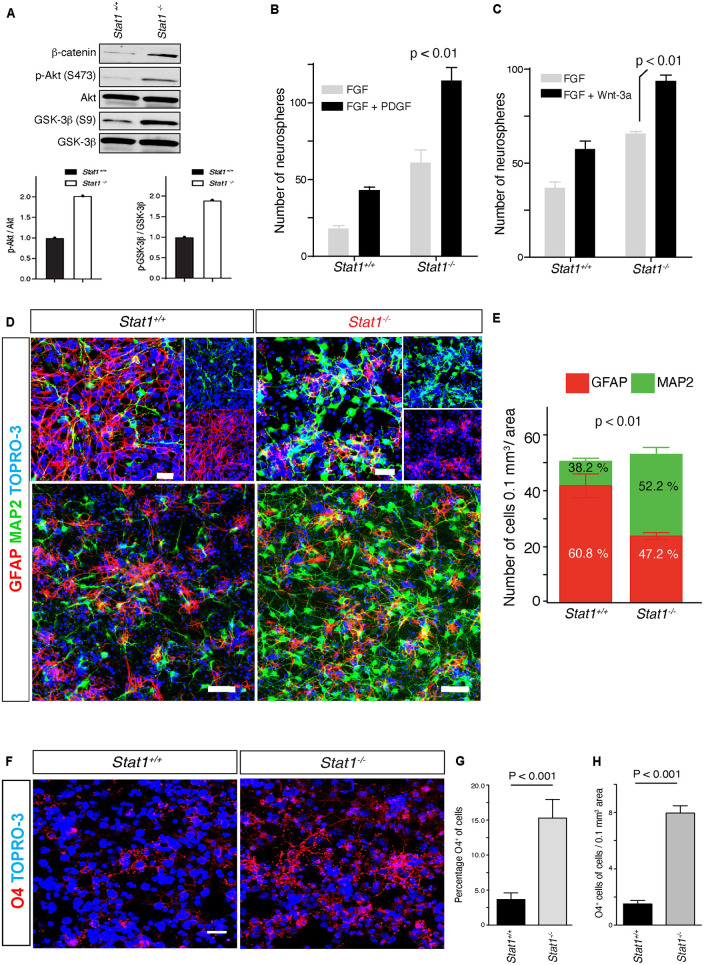
*Stat1*-deficient NSCs exhibit increased responses to SVZ self-renewal signals and differentiation *in vitro*. **(A)** Western blot and quantification of β-catenin expression, Akt phosphorylation and GSK-3β activation from *Stat1*^–/–^ vs *Stat*^+/+^ NSCs. **(B,C)** Quantification of self-renewal response in the presence of PDGF-β and Wnt3 in *Stat1*^+/+^ and *Stat1*^–/–^, NSCs from *Stat1*^+/+^ and *Stat1*^–/–^ mice, there is an increase response in *Stat1*^–/–^ NSCs *p* < 0.01 **(D)** Confocal microscopy of differentiation of neurospheres from *Stat1*^+/+^ and *Stat1*^–/–^ stained with (MAP2 (*green*) and GFAP (*red*) there is an increased in the numbers of MAP2 positive cells in the *Stat1*^–/–^ NSCs cultured in NSCs differentiation medium. Top panel: higher magnification Lower panel: Lower magnification of different area. **(E)** Quantification of numbers of MAP2 and astrocytes in differentiating NSCs from *Stat1*^+/+^ and *Stat1*^–/–^ showing an increased in the numbers of differentiated progenitors with increased in neurogenesis *p* < 0.001 by *t-test*. Bar: 50 μm *(n* = *3*, representative of 3 experiments). **(F)** Confocal microscopy of differentiation of neurospheres from *Stat1*^+/+^ and *Stat1*^–/–^ stained with O4 (*red*) and TOPRO-3 (blue) there is an increased in the numbers of O4 + positive cells in the *Stat1*^–/–^ NSCs cultured in NSCs differentiation medium. **(G,H)** Quantification of numbers of O4 + progenitors in differentiating NSCs from *Stat1*^+/+^ and *Stat1*^–/–^ showing an increased in the numbers and percentage of differentiated oligodendrocyte progenitors *p* < 0.001 by *t-test*. Bar: 10 μm.

*In vivo*, NSCs respond to niche signals to maintain self-renewal capacity. Our *in vivo and in vitro* data suggest that *Stat1*-deficient NSCs may respond differentially to known SVZ niche cytokines (Jackson et al., 2006; Kalani et al., 2008). To investigate the responses of NSCs from *Stat1*^–/–^ mice to niche cytokines, we exposed the isolated *Stat1*^–/–^ NSCs to self-renewal signals *in vitro*. In response to EGF and FGF-2, *Stat1*^–/–^ NSCs exhibited more stabilized β-catenin, increased Akt phosphorylation, and GSK-3β inactivation demonstrated by increased phosphorylation GSK-3β at serine 9 (S9) (Figure 3A). Next, we tested the responses of *Stat1*^–/–^ NSCs to PDGF-β (Jackson et al., 2006) and Wnt-3a (Kalani et al., 2008), both of which increase self-renewal and neurogenesis in the SVZ (Kalani et al., 2008; Favaro et al., 2009). In response to these cytokines, *Stat1*^–/–^ NSCs increased the number of secondary neurospheres (*p* < 0.05) (Figures 3B, C), indicating that the machinery important for SVZ cytokine-induced self-renewal by PDGF and Wnt3 is not only functional, but increased in *Stat1*^–/–^ NSCs in response to PDGF-β and Wnt-3a.

Next, we studied the differentiation of NSCs in the absence of *Stat1* by plating similar number of NSCs in PLL-coated dishes in neurobasal differentiation medium that halts proliferation and initiates differentiation. Seven days after plating, we counted the number of newborn neurons and differentiating astrocytes in culture. *Stat1* deletion induced a relative increase in the number of MAP2 + neurons and reduced the number of GFAP + astrocytic cells (*p* < 0.05) (Figures 3D, E) compared to controls as shown by confocal. We confirmed this increased neurogenesis by staining for doublecortin (a marker of neuroblasts) and found a statistically significant increase in doublecortin-positive cells (*p* < 0.05) (Supplementary Figures 2A, B). We also observed an increase in O4 + oligodendrocyte precursor percentage and numbers per area in the *Stat1-*deficient NSCs (*p* < 0.001) (Figures 3F–H). Together, these data suggest *Stat1* negatively regulates neuronal and oligodendroglia differentiation.

### *Stat1* is induced in the SVZ during EAE and correlates with increased presence of IFN-γ-producing T cells in the meninges and cerebrospinal fluid (CSF)

Aging is associated with inflammation of the SVZ niche characterized by the activation of *Stat1* signaling on SVZ NSCs and the presence of T cells, suggesting that aging induces inflammation of the SVZ niche mediated by IFN-γ-producing T cells (Dulken et al., 2019). T cell cytokines in the cerebrospinal fluid (CSF) may target NSCs, since these cells (type B NSCs) protrude part of their cytoplasm into the ventricle (Lehtinen et al., 2011; Warre-Cornish et al., 2020; Supplementary Figure 3A). Consistent with this, we found that interferon-γ-associated genes are expressed in the NSCs in the SVZ (Supplementary Figures 3B, C), consistent with the role of IFN-γ in the aging SVZ (Dulken et al., 2019).

**FIGURE 4:**
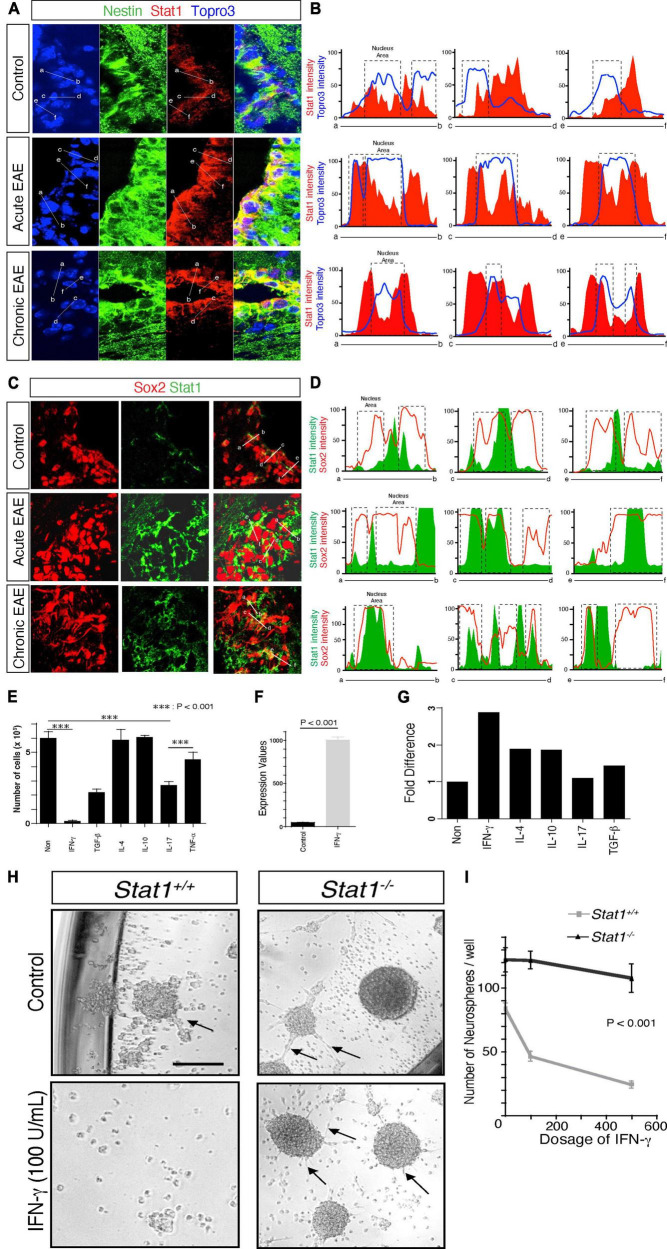
Increased *Stat1* mediates IFN-γ alteration of self-renewal in NSCs. **(A)** Expression of STAT1 in nestin + positive NSCs from the SVZ in control, acute and chronic EAE in SJL mice. **(B)** Confocal quantitative pixel measurements of STAT1 in nestin + NSCs, indicating cytoplasmic and peri and nuclear increase of STAT1. **(C)** Expression of STAT1 in Sox2 positive NSCs from the SVZ in control, acute and chronic EAE in SJL mice. **(D)** Confocal quantitative pixel measurements of Stat1 in Sox2 positive NSCs, indicating an increase in STAT1 in cytoplasmic and peri and nuclear areas, Sox2 is highly expressed in the nucleus and colocalize with STAT1. **(E)** Quantification of proliferation of NSCs exposed to various cytokines, quantified at day 7 by thymidine incorporation, *n* = *3*. *p* < 0.0001 by *t-test.* **(F)** Expression of *Stat1* by PCR of NSCs derived from the SVZ after exposure to IFN-γ 100 u/ml **(G)** Quantitative PCR analysis of *p16*^/ink4^ after exposure to cytokines. there is a threefold increase in the expression of *p16*^/ink4^ after exposure to IFN-γ and other cytokines **(H)** Microscopy of cultured neurospheres from *Stat1*^+/+^ and *Stat1*^–/–^ SVZ cells in the absence or presence of 100 U/ml IFN-γ. **(I)** Quantification of neurospheres from *Stat1*^–/–^ and *Stat1*^+/+^ mice exposed to IFN-γ. NSCs from *Stat1*^+/+^ exhibited a reduction of the numbers of neurospheres that is dose dependent compared to *Stat1*^–/–^ NSCs that are resistant to the effects of IFN-γ reduction in size and numbers, and the colonies were smoother with more spontaneous formation of chain migration (arrows) (*p* < 0.001) *n* = 4 independent experiments. Bar: 100 μm.

Next, we asked if *Stat1* is likewise activated under disease conditions, such experimental autoimmune encephalomyelitis (EAE), a model of MS. We have previously found that *Stat1* is increased in the SVZ in MOG-EAE (Pluchino et al., 2008). To confirm these observations in a different EAE model, we first examined *Stat1* gene expression in the isolated SVZ SJL mice showing a neurological score of >2. In these mice, *Stat1* increased two-fold and nine-fold in the SVZ during the acute (day 15) and chronic phases of EAE (day 30 post-immunization) (Supplementary Figure 3D). To confirm these gene expression observations at the protein level, we used NSC-specific markers. We found increased expression of STAT1 protein in Nestin + NSCs specifically in NSC cytoplasm and nuclear regions, by using quantitative confocal imaging with pixel measurements during EAE in the PLP 139–151 model *in vivo* (Figures 4A, B). We confirmed these findings by using Sox2, a marker for SVZ neural stem and progenitor cells repeating the quantitative confocal measurements showing similar findings (Figures 4C, D).

Next, we hypothesized that increased STAT1 signaling in our EAE SJL model may correlate with the presence of infiltrating T cells. To test this, we measured the frequency of IFN-γ^+^ and IL17^+^ CD4 + cells in the meninges and ventricular CSF obtained from SJL mice. There was a significant increase in the number of IFN-γ-producing cells during the acute and chronic phases of EAE compared to IL-17 producing cells (*p* < 0.001) in the CSF and meninges (Supplementary Figure 3E). To confirm this observation as a broader pathological mechanism in EAE, we used MOG EAE adoptive transfer. We already demonstrated that *Stat1* is increased in the SVZ of EAE mice (Pluchino et al., 2008). To correlate this increased expression with IFN-γ + producing cells, we measured the frequency of antigen-specific IFN-γ + and IL-17 + cells in the meninges and CSF by adoptive transfer of 2D2 transgenic cells in mice with significant clinical scores > 2. The frequency of MOG-specific T cells producing IFN-γ was significantly higher than of IL17 + transgenic cells, suggesting that IFN-γ is a predominant cytokine affecting the SVZ niche in EAE in both models (Pluchino et al., 2008; Rasmussen et al., 2011; Supplementary Figure 3F).

### An interferon-γ-inducible *Stat1* program mediates decreased NSC self-renewal

Despite the role of IL-17 producing cells in the pathogenesis of EAE (Kebir et al., 2007; Steinman, 2007), our observations suggest that IFN-γ may play a more prominent role on NSCs *in vivo.* To investigate this further, we exposed NSCs to a panel of cytokines *in vitro* for 7 days and measured their number. We found that IFN-γ suppressed the proliferation of NSCs significantly more than IL-17 (*p* < 0.0001) (at the standard effective doses of both cytokines) or other cytokines such as IL-10 and IL-4 (Figure 4E). At these dosages, IFN-γ increases the expression of *Stat1* and *p16* in NSCs associated with cell cycle arrest (Figures 4F, G). Next, we extended these *in vitro* observations by examining the exposure to IFN-γ in NSC self-renewal. First, we found a dose-dependent decrease in the self-renewal capacity of NSCs at 100 U/ml, when STAT1 phosphorylation was activated (Supplementary Figures 4A, B). IFN-γ did not affect the levels of apoptosis (Supplementary Figure 4C), a result consistent with effects on proliferation that has been shown before (Pereira et al., 2015). In addition, there was an increase in Tapasin-1 (an interferon gene involved in MHC class I function) and downregulation of tenascin (a gene involved in stem cell renewal) (Garcion et al., 2004; Supplementary Figures 4D, E). These mRNA changes were reflected at the protein level. Tenascin-C was downregulated by IFN-γ (*p* < 0.008) and Tapasin (*p* < 0.008) was upregulated by IFN-γ at the protein level as revealed by confocal microscopy (Supplementary Figure 4F). More notably, we cultured early and late passage NSCs under IFN-γ for 9–12 weeks to mimic a chronic neuroinflammatory microenvironment and observed a decrease in the population doubling with lack of formation of neurospheres more pronounced in younger NSCs than late culture NSCs indicating that despite *in vitro* adaptation NSCs were susceptible to IFN-γ. This effect was partially reversed after IFN-γ was withdrawn from the culture. These data suggest an effect of IFN-γ on NSC lifespan that persists even in the absence of additional IFN-γ (Supplementary Figures 4G, H). To determine the specificity of *Stat1* in mediating the effects of IFN-γ, we cultured NSCs from *Stat1*^–/–^ and WT with IFN-γ. NSCs from WT cultured with IFN-γ showed a dose-dependent reduction of self-renewal capacity (Figure 4H) while *Stat1*^–/–^ NSCs were resistant to IFN-γ (*p* < 0.001, of note the neurospheres in the *Stat1*^–/–^ were smoother with more spontaneous formation of chain migration of neural progenitors (Figure 4I).

### *Stat1-deficient* NSCs increases the expression of genes for self-renewal and differentiation

**FIGURE 5:**
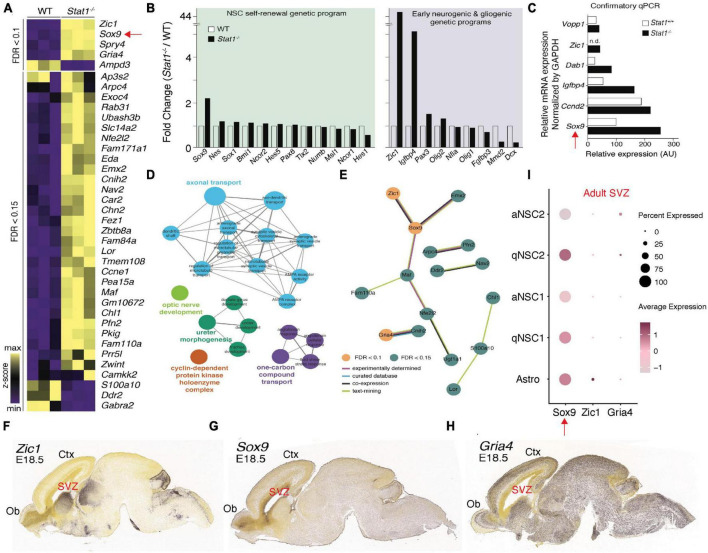
Differential expression of self-renewal and neurogenic genes following *Stat1* deletion. **(A)** Heatmap of differentially expressed genes (DEGs) between *Stat1*^+/+^ and *Stat1*^–/–^ NSCs at FDR < 0.1 and 0.15, respectively. **(B)** Fold change (*Stat1*^–/–^*/Stat1*^+/+^) of genes underlying NSC self-renewal, and early neurogenic and gliogenic programs. **(C)** Plot of confirmatory PCR values in *Stat1*^+/+^ and *Stat1*^–/–^ NSCs for selected genes using GADPH as control **(D)** Gene ontology enrichment for DEGs at FDR < 0.15. Node sizes reflect level of statistical significance and are colored by group. **(E)** PPI network of DEGs with only interacting proteins being shown. Nodes are colored by FDR threshold and edges by evidence source. Data derived from StringDB. **(F–H)** *In situ* hybridization images of the developing mouse brain at E18.5 with probes for **(F)** *Zic1*, **(G)** *Sox9*, and **(H)** *Gria4* from the Allen Brain Atlas. **(I)** Single cell RNAseq Dot plot for *Sox9*, *Zic1*, and *Gria4* expression in the adult SVZ by cell type indicating high expression of *Sox9* in all SVZ NSCs and SVZ astrocytes.

To determine the molecular impact of *Stat1* deletion as a model for a potential therapeutic intervention on NSCs, we performed microarray analysis on WT and *Stat1*^–/–^ NSCs. Despite being a transcription factor, *Stat1* deletion induced a limited differential gene expression in NSCs (Figure 5A). Among the top differentially expressed genes (DEGs), we found high expression of *Sox9* (important in NSC self-renewal) and *Zic1* (important for neurogenic differentiation) (Figure 5B). Confirmatory qPCR validated our microarray results for several of these genes with *Sox9* being the top upregulated gene (Figure 5C). Gene ontology analysis for DEGs showed an enrichment in cell cycle regulation, dentate gyrus development, where adult neurogenesis occurs, and neuronal processes, like axonal transport (Figure 5D). We next performed protein-protein interaction (PPI) analysis on DEGs, we found our PPI network to include significantly more interactions than expected (*p* = 0.00383), suggesting these DEGs are biologically connected including Zic1-Sox9 (Figure 5E). Finally, we explored the location of the increased gene expression signature using the Allen Gene Expression Database^1^ to confirm the regional and temporal expression of increased genes in *Stat1* deleted cells to NSC areas by *in situ* hybridization patterns. This data was available in the developing embryonic mouse brain. While *Gria4* showed predominant expression in the cortical plate of the expanding cortex, *Zic1* and *Sox9* expression localized to areas of NSC proliferation, such as the SVZ and olfactory bulb (Figures 5F-H). To examine their expression in the adult mice SVZs, we leveraged the scRNA-seq dataset of adult SVZ NSCs (Kalamakis et al., 2019). Among the differential expressed genes, we found high levels of *Sox9* expression across all NSCs types including astroglia cells in the SVZ (Figure 5I).

### *Stat1* transcriptionally represses *Sox9*

**FIGURE 6:**
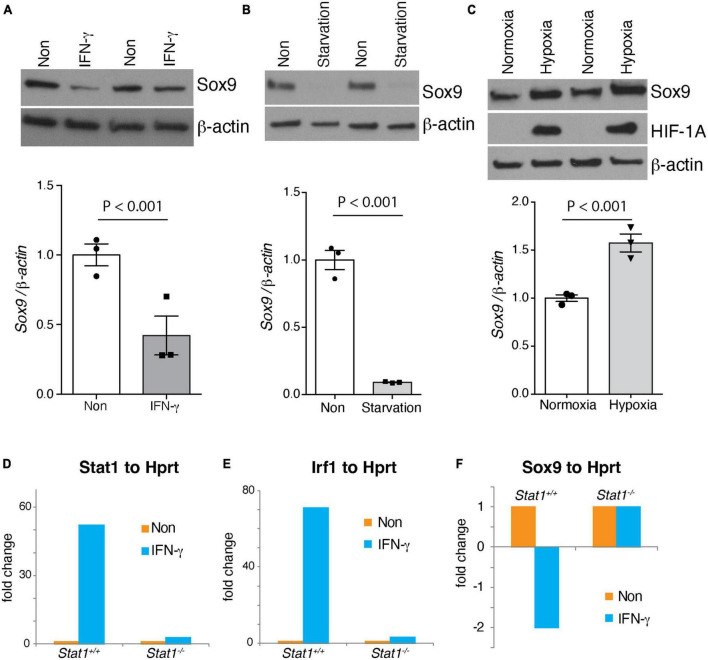
Regulation of Sox9 NSCs and *Stat1* dependency of IFNγ effects. Western blot (top) of physiological and pathological signals regulating the SVZ, shown as technical duplicates. **(A)** IFNγ **(B)** NSCs starvation **(C)** and hypoxia differentially regulates Sox9 levels. (Bottom) shows quantification of Sox9 protein levels using β-actin as control, showing statistical significance differences. IFNγ and starvation of NSCs reduces Sox9 levels, while hypoxia increase Sox9 expression **(D,E)** *Stat1* dependency of the increase of *Stat1* and *Irf1* gene expression by IFNγ, **(F)** *Stat1* dependency of the reduction of *Sox9* expression by IFNγ in NSCs.

**FIGURE 7:**
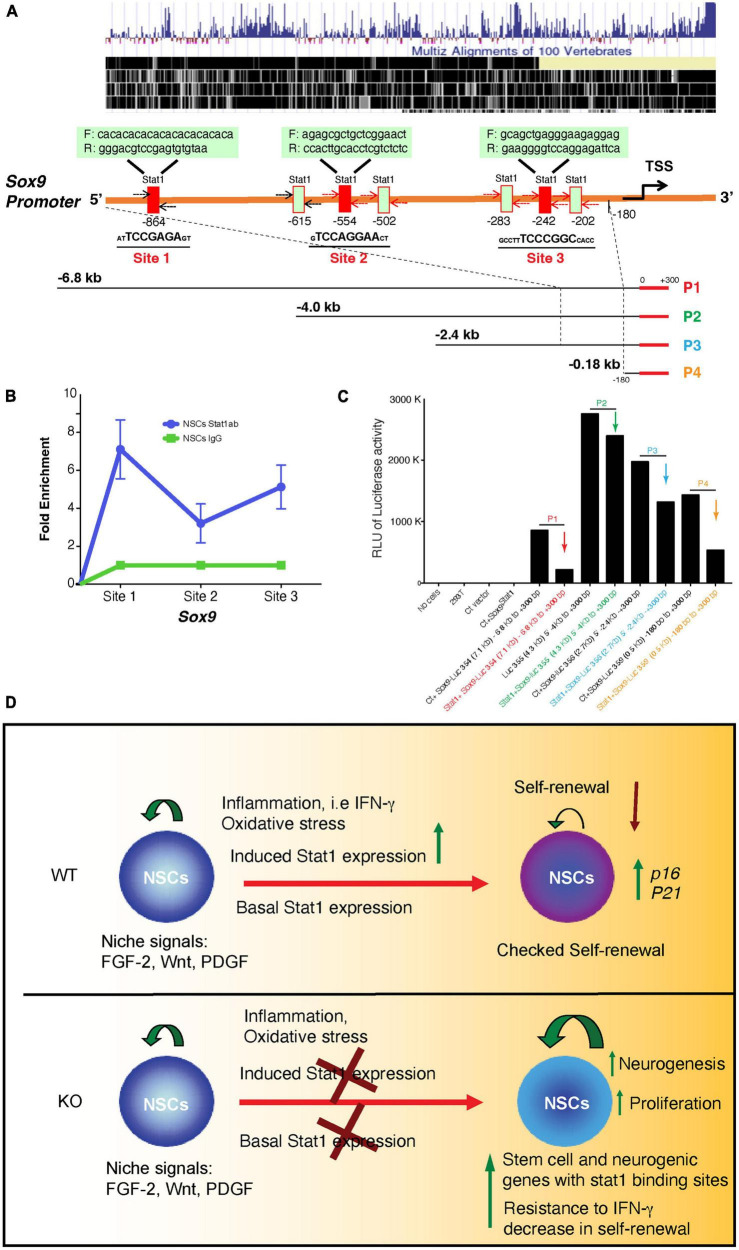
Direct interaction of *Stat1* and *Sox9* and transcriptional repression in a luciferase assay and model. **(A)** Conserved map of the endogenous sox9 promoter among vertebrates and Schema of positions of seven and putative *Stat1* binding sites within the mouse *Sox9* promoter. Three candidate binding sites were selected (underlined). Primer sets used in the PCR assays for the detection of *Stat1* binding to the *Sox9* promoter using the ChIP products are shown. **(B)** ChIP analysis of *Stat1* binding to the three sites of the *Sox9* promoter. Antibodies used for IP are anti-Stat1 and control IgG. Total input DNA before IP was used for normalization of data. Data are represented as fold enrichment. **(C)** Luciferase assay of four different *Sox9* promoter regions targeted by *Stat1* construct (P1-P4) demonstrating transcriptional repression. HEK 293T cells were transfected with a constant amount of four different *Sox9* promoter-luciferase plasmids encoding four different regions of the *Sox9* promoter as indicated (P1, P2, P3, and P4) with or without *Stat1* vectors. Cells were lysed 24 h. later and luminescence was measured, data is representative of three independent experiments. **(D)** Model of the role of *Stat1* in NSCs during physiological and neuroinflammation conditions, in the presence of chronic inflammation (i.e., chronic MS), oxidative stress and inflammatory cytokines (i.e., IFN-γ) upregulate STAT1 expression in NSCs leading to decreased self-renewal capacity, in addition to increase in senescence genes such as *p16* and decreased expression of stem cell genes such as *Tnc, Sox9* (*top panel*) as shown in our data. In the absence of *STAT1*, the NSCs self-renewal capacity is increased and deletion increased neurogenesis and self-renewal and neurogenic gene expression (our data). During inflammation (i.e., IFN-γ increased) *STAT1* deletion may associated with resistance to IFN-γ deleterious effects.

*Sox9* is a well-known transcriptional regulator of self-renewal in NSCs (Scott et al., 2010). However, the signals that regulate the expression of *Sox9* in NSCs are unknown. We study whether physiological and pathological states regulate the expression of *Sox9* in NSCs. Indeed, we find that Sox9 expression positively correlates with signals such as hypoxia associated with increased self-renewal. On the contrary, pathological signals associated with decreases self-renewal such as IFN-γ and starvation decreases Sox9 expression (Figures 6A-C). Furthermore, we investigated whether the expression of *Stat1* target genes like *Stat1* and *Irf1* where dependent of *Stat1*, we showed that these genes are dependent of the presence of *Stat1* in NSCs (Figures 6C, D). Next, we asked for the mechanisms of *Stat1* regulation of *Sox9* in NSCs. Because of the upregulation of *Sox9* upon *Stat1* depletion, we hypothesized *Stat1* may directly regulate *Sox9* expression. To test this, we performed qPCR for *Sox9* in WT and *Stat1*-deficient cells response to IFN-γ. We found that in the presence of IFN-γ, *Sox9* only was downregulated in WT cells, but not *Stat1*-depleted cells, suggesting that *Stat1* may directly regulate *Sox9* at the transcriptional level (Figure 6F). To test this hypothesis, we searched the *Sox9* promoter for potential binding sites for *Stat1* using the Biobase database. We identified overlapping putative binding sites for *Stat1* upstream of the transcription start site (TSS) of Sox9 in highly conserved area among vertebrates (Figure 7A). We validated three putative sites of potential binding of Sox9 as a direct target of *Stat1* in NSCs by performing chromatin immunoprecipitation followed by qPCR (ChIP-qPCR) in NSCs exposed to IFN-γ. Primer sets flanking the *Stat1* binding at three sites (Site1-3) in the Sox9 promoter were designed to amplify the immunoprecipitated ChIP DNA by qPCR. We observed a significant increase in *Stat1* binding to these three sites in the *Sox9* promoter in NSCs exposed to IFN-γ (Figure 7B). To analyze the functional relevance of the binding of *Stat1* to their target sequences in the *Sox9* locus, we investigated the ability of *Stat1* to regulate the activity of the *Sox9* promoter in reporter assays. We used four reporter constructs pGL3-*Sox9* containing the firefly luciferase gene under the control of the *Sox9* promoter. It should be noted that these vectors span multiple conserved genomic regions in *Sox9* that are highly conserved among Human, Rhesus, Mouse, and Dog that include all our binding sites for *Stat1*. Notably, we found that *Stat1* was able to transcriptionally repress all the *Sox9*-luciferase constructs, especially a highly conserved region near the transcriptional start site that is highly conserved during evolution (Figure 7C).

## Discussion

A central unanswered question in regenerative neuroimmunology is the mechanisms by which NSCs are activated or inactivated to initiate or halt repair during disease and how this process changes over time (Poss, 2010). Identification of the genes and molecules differentially induced by acute versus chronic states is a critical step toward developing therapies for lasting neural regeneration. Complementary to the use of exogenous NSCs, the investigation into the pathways that control endogenous NSCs during repair is more limited. For instance, the search for genes that control self-renewal capacity of stem cells like NSCs has largely focused on cell type-specific enriched genes through comparative transcriptome analysis between diverse populations of stem cells and adult tissue (Ramalho-Santos et al., 2002; Suslov et al., 2002). This approach has been successful in identifying genes that, by nature of their high expression in NSCs, have a dominant function during development and adult homeostasis. However, this approach overlooks inducible genes and programs in NSCs activated only during disease states that can change the NSCs internal molecular programs at the genetic and epigenetic level.

Our study provides new evidence of the mechanistic role of the IFNγ-receptor-Stat1 pathway in SVZ-derived NSCs in neuroinflammation. New data in pathological situations like aging involves Type II interferon in NSC modulation by T cells (Dulken et al., 2019), but the interplay between Type I and II interferon during normal and pathological states and SVZ function is still unclear. The situation during pathology — especially neuroinflammation models — may be different. For instance, in EAE there is a profound alteration in the SVZ cells that leads to a reduction of NSC self-renewal *in vivo* and *in vitro* with reduction in neurogenesis in chronic EAE; this has been shown by our group and others (Pluchino et al., 2008; Rasmussen et al., 2011; Tepavčević et al., 2011). There are major transcriptional changes in the SVZ at the molecular level during inflammation in EAE that generate sustained disease-associated programs in the SVZ in chronic stages of the disease. These changes induced by chronic inflammation are orders of magnitude higher compared to the physiological function of interferons in the normal SVZ (Starossom et al., 2011, 2019). Therefore, Type II interferon may change the transcriptional landscape of the SVZ with aberrant and sustained molecular programs by chronic exposure.

Our data show that NSCs self-renewal capacity can be modulated by IFNγ, a cytokine from the inflamed environment that induces an abnormal transcriptional program in NSCs, including an IFNγ receptor-Stat1-Sox9 pathway. By taking such an approach to identify inducible molecules, we find that during normal homeostasis *Stat1* is a negative regulator of NSCs in response to normal niche molecules that may include small amounts of IFN-γ, as shown previously (Li et al., 2010). However, during inflammation such as in EAE, IFN-γ increases the *Stat1* transcriptional program and results in increased *p16*^/ink4*a*^ and *Stat1*, leading to decreased self-renewal and lifespan that is sustained and changes these cells’ repair capacity. In our model, IFN-γ changes the transcriptional landscape of NSCs with sustained molecular programs as shown in our *in vitro* experiments. In culture, IFN-γ has a sustained effect that alters the lifespan and self-renewal of NSCs. Using *Stat1-*deficient NSCs, we showed that the effects of IFN-γ are mediated in part by the transcriptional function of *Stat1*, results that could have translational relevance in the protection of NSCs from the inflamed microenvironment. More, notably we find that *Stat1*-deficient cells may have increased neurogenesis and oligodendrogenesis associated with an increase in *Sox9*, findings that have potential translational implications but need to be better defined *in vivo*. Finally, we demonstrate that *Stat1* binds and modulates the function of *Sox9*. We summarize this simplified model of the role of *Stat1* in NSCs in Figure 7D. As just one example, future genome-wide studies will be critical for defining other direct transcriptional targets of *Stat1* in NSCs.

Our study confirms the functional role of *Stat1* in regulating NSC function *in vitro*. Previous work identified the specific role of *Stat1* in Nestin + progenitors from the SVZ by reducing *Stat1* dependent NSC proliferation; this was confirmed with our data (Pereira et al., 2015). Furthermore, we expanded the role of *Stat1* in NSCs by focusing on the impact of *Stat1* in NSC self-renewal and differentiation *in vitro*. Future work will be needed to further delineate the role of *Stat1* in neuroblast formation *in vivo*, since it has been shown that IFN-γ increases the number of neuroblast (Pereira et al., 2015), and our NSCs deficient in *Stat1 in vitro* show an increase in differentiation of neurons and neuroblast. Differences in methodology may explain the difference between these findings. For instance, emerging data highlight that aging and immune-related inflammation overlap in many of their molecular targets. T cells increasingly infiltrate the aging SVZ and interact with resident NSCs to temper adult neurogenesis through interferon stimulated gene (ISG) signaling (Dulken et al., 2019). This is similar to our results in EAE that show in the SJL and MOG-EAE models a reduction in olfactory bulb neurogenesis (Pluchino et al., 2008; Rasmussen et al., 2011) that has been confirmed by others (Tepavčević et al., 2011).

There is recent interest in defining the impact of interferons in the SVZ, and recent work has shown an important role of interferons in regulating SVZ NSCs in young and old mice. Type I interferon modulates the activation of SVZ NSCs and induces quiescence (Kalamakis et al., 2019; Carvajal Ibañez et al., 2023). At the same time, other groups report major contributions of type I interferon signaling in the aging choroid plexus and ventricular zones (Baruch et al., 2014). A crucial next step will be to disentangle the contributions and interplay between these different types of interferons. In addition, investigation into the role of ISG signaling in other neural stem cell niche areas like the hippocampus and aging-associated diseases like dementia could be critical for repair in neurodegeneration. Similar mechanisms of dysfunction of a stem cell niche are reported in the bone marrow where loss of normal signaling in the niche can precipitate disease (Raaijmakers et al., 2010) or exogenous signals such as immune cytokines can affect the normal regulation in the niche, leading to bone marrow failure (de Latour et al., 2010; Tang et al., 2010).

Despite the success in animal models of MS and promise from a recently completed open-label, phase one study showing the safety of intrathecal infusions of fetal-derived NSCs (Genchi et al., 2023), infusions of NSCs to MS patients face many hurdles. These questions include the sourcing of stem cells; the use of fetal allogeneic vs. induced pluripotent stem cells; the safety of cultured human stem cells such as NSCs (Imitola, 2007) or embryonic stem cells (Gore et al., 2011); their potential for tumorigenicity (Snyder, 2011); and whether exogenous NSCs may be compromised when placed in a chronically-inflamed microenvironment, becoming the target of neuroinflammation. Therefore, understanding the mechanisms by which endogenous stem cells fail to repair during disease and the dissection of inducible aberrant transcriptional programs by cytokines critical for MS, like IFN-γ in collaboration with other cytokines like TNFα and IL17 among others, thus remains of paramount importance.

Limitations of the current work includes a detail analysis of the relationship of *Stat1* in multiple cell types including *Dlx2, DCX* neural progenitors in the SVZ. Future single-cell sequencing identification of such neuroinflammation-induced programs of NSCs in the SVZ *in vivo* will be a critical next step to expand the distinct molecular programs in NSCs and the role of other inflammatory cells such as T cells and microglia (Starossom et al., 2011, 2019).

In conclusion, our data expand of the role IFN-γ on NSCs and suggest that the transcriptional programs of *Stat1* may regulate NSCs and other progenitors in the SVZ. Identifying the cell type-specific genetic networks that respond to injury and inflammation, such as *Stat1*, will be key to rationally designing pro-regenerative therapies in chronic progression of neuroinflammatory diseases such as MS.

## Experimental methods

### Animals

We used 6–8 weeks C57BL/6, and SJL mice obtained from The Jackson Laboratory (Bar Harbor, Maine, USA). MOG-Tg 2D2 mice were provided by Dr. Vijay K. Kuchroo. 6–8 weeks old 129S6/SvEv-*Stat1*^*TM*1*Rds*^ mice containing a homozygous disruption of the *Stat1* gene and complete lack of functional STAT1 proteins were obtained from Taconis Bioscience (Germantown, NY, USA). All mice were housed according to NIH guidelines and the Animal Care Committee of Harvard University approved all experiments. All NSCs experiments were done according to the regulation of the University of Connecticut Biosafety regulations.

### EAE induction

All experiments were conducted under the approval of the Harvard Medical School Animal Care committee and the Animal Care. SJL/J mice were immunized subcutaneously in two sites (left and right flank) with 150 μg of PLP139-151 (New England Peptide LLC., Gardner, MA) emulsified in complete Freund’s adjuvant (CFA, Sigma Aldrich, Saint Louis, MO) containing 200 μg Mycobacterium Tuberculosis (Difco Laboratories, Detroit, MI). Mice received 200 ng pertussis toxin (PT, List Biological Laboratories Inc., Campbell, CA) in 0.2 ml PBS (Lonza, Walkersville, MD) intraperitoneally (ip) at the time of immunization and 48 h later. Control mice were immunized with CFA followed by PT (12). The mice were sacrificed at different time points. For MOG 35–55 (M-E-V-G-W-Y-R-S-P-F-S-R-O-V-H-L-Y-R-N-G-K) corresponding to mouse sequence is synthesized by QCB Inc. Division of BioSource International (Hopkinton, MA), and purified to > 99% by HPLC. Mice are immunized subcutaneously in the flanks with 150–200 μg of MOG peptide in 0.1 ml PBS and 0.1 ml CFA containing 0.4 mg Mycobacterium Tuberculosis (H37Ra, Difco, Detroit, Michigan) and injected i.p. with 200 ng Pertussis toxin (List Laboratories, Campbell, California) on the day of immunization and 2 days later. EAE is scored as follows; grade 1, limp tail or isolated weakness of gait without limp tail; grade 2, partial hind leg paralysis; grade 3, total hind leg or partial hind and front leg paralysis; grade 4, total hind leg and partial front leg paralysis; grade 5, moribund or dead animal. All animals used for immunohistochemistry experiments of acute and chronic EAE, had a clinical score of equal or > 2. For adoptive transfer, we collected 3 × 10^6^ CD4 + MOG TCR transgenic cells from a naïve 2D2 mouse and injected iv into 4 WT females. The recipients were immunized with 150 μg MOG/CFA + PT and harvested at day 14 pi. EAE scores were 2–3. The samples were stimulated with PMA and ionomycin for 4 h before staining. MOG TCR transgenic cells were identified based on their homogenous expression of TCR Va3.2 and Vb11.

### Neural stem cell isolation and culture

Primary culture of adult neural stem cells. Primary cultures of neural stem cells were isolated from adult 129 *Stat1*^+/+^ and *Stat1*^–/–^ mice. Mice were deeply anesthetized in a CO_2_ chamber at the endpoint of the various time points. Two mm coronal sections were taken from the brains and periventricular tissue was dissected into cold HBSS. The tissue was cut into small pieces (>0.1 mm) and transferred to a digestion solution (mixture of papain, cysteine and EDTA). At the end of the enzymatic incubation the tissue was mechanical dissociated with a fire-bore narrowed Pasteur pipette in DMEM/F-12 medium containing 0.7 mg/ml ovomucoid. The dissociated cell suspension was plated in non-coated flasks in media composed of DMEM/F-12, N-2 supplement (1%), penicillin/streptomycin (1%), bFGF (20 ng/ml), EGF (20 ng/ml) and heparin (8 μg/ml). Primary stem cell proliferation was detected after 7 days *in vitro* and characterized by formation of spheres of undifferentiated cells. Subsequently passaging of primary spheres was performed by enzymatic incubation with versene (5 min at 37°C) and mechanically dissociating the collected spheres.

The dissociated cell suspension was then again plated in non-coated flasks for secondary neurospheres stem cell proliferation. Before each experiment NSCs were dissociated with versene and plated in PDL-coated 24 well-plates (1*10^5^ cells/ml) in DMEM/F-12 medium for 24 h whereafter the medium was changed to neurobasal medium supplemented with B27 (1%), L-glutamine (0.5 nM) and penicillin/streptomycin (1%) for differentiation.

For differentiation of NSCs from *Stat1*^+/+^ and *Stat1*^–/–^ NSCs were plated at 1 × 105 cells/ml in 24 well plates in neurobasal medium supplemented with B27 (1%), L-glutamine (0.5 nM) and penicillin/streptomycin (1%) for differentiation that stop the proliferation of NSCs and allow neuronal and glial differentiation after 7–14 days *in vitro*. At the end of the differentiation period cultures were placed on PFA4% in PBS for staining with antibodies for neurons (MAP2, DCX), astrocytes (GFAP) and oligodendrocyte progenitor cells (O4).

#### Culture of NSCs with cytokines, continuous growth curve, and population doublings

To culture NSCs with cytokines for proliferation assay, NSCs were cultured for 7 days with IFNγ 100 u/ml, IL17, 10 ng/ml. TGF-β, 1 ng/ml, IL4, 1 ng/ml, IL10, 1 ng/ml in 96 well plates and processed for thymidine incorporation to determine proliferation.

For continuous growth and population doubling. 3 × 10^5^ NSCs were be plated in T75 flasks with or without IFN-γ at 100 U/ml and allowed to proliferate. On day 3, the cells are counted and 3 × 10^5^ of the live NSCs are re-plated under the same conditions. The numbers of population doublings are calculated using a standard formula: PD = ln(Nf/Ni)/ln 2. Where PD = Population doubling, Ni = is the initial amount of NSC, Nf = final amount of NSCs per passage, ln = natural logarithm. Population doubling is calculated for a total of 5–10 passages (Jacobs et al., 2000). This experiment allow us to establish whether the continuous presence of IFN-γ at non-apoptotic dosages is sufficient to generate a senescent phenotype in the NSCs *in vitro*. This approach was used to compared *Stat1*^–/–^ and *Stat1*^+/+^ NSCs.

#### *Stat1* retrovirus infection and self-renewal neurosphere assay

The details from the retroviral virus and infections have been provided, STAT1 signaling was increased in NSCs by transduction with a murine stem cell virus–based (MSCV-based) retroviral vector as previously described and received from G. Lesinski (Lesinski et al., 2003). A brief description is provided, the genome of this virus contains 5′ and 3′ long terminal repeats (LTRs), a multiple cloning site, and an internal ribosomal entry signal (IRES) followed by the enhanced green fluorescent protein (EGFP) coding region. A plasmid containing the STAT1 coding sequence was obtained and the STAT1 sequence was excised with *Sac*II, blunted with Klenow fragment DNA polymerase, and digested with *Bam*HI to generate a sticky end 5′ to the STAT1 coding sequence. This strategy permitted the directional cloning of the STAT1 sequence into the *Bam*HI/*Hpa*I-digested MSCV retroviral genome. The genes encoding STAT1 and EGFP are expressed as a single transcript, therefore a marker of Stat1 gene overexpression. To generate retroviral supernatants, 293T cells were transiently transfected by calcium phosphate–mediated coprecipitation as previously described. Briefly, 1.5 × 10^7^ 293T cells plated overnight in 15-cm dishes with polylysine (Sigma-Aldrich) were cotransfected with plasmids encoding the gag and pol proteins from the Moloney murine leukemia virus (M-MLV) (25 μg), coat proteins from the vesicular stomatitis virus (VSV) (5 μg), and the STAT1-IRES-EGFP construct or a control construct (MSCV) lacking the STAT1 sequence (25 μg). The retroviral supernatant was harvested at 48 h after transfection, filtered (0.2 micron), and frozen at −70°C (23). NSCs cells as neurospheres for infection were washed, trypsinized, seeded in a 6-well plate at 5 × 10^5^ cells per well. For infection, the culture medium was removed, and 1 ml of thawed retroviral supernatant was added per well in the presence of polybrene (8 μg/ml final concentration). Plates were spun for 1 h at 1,000 × *g*, incubated overnight at 37°C with 5% CO_2_, and supplemented with new media the next day. Cells were assayed 48 h after infection by flow cytometric analysis for EGFP expression using uninfected NSCs for comparison. EGFP-positive cells were sorted into a 96-well plate as single cell clones.

The number of neurospheres formed in the presence of FGF-2 divided by the number of cells plated gives the neurosphere frequency (Molofsky et al., 2003). For self-renewal capacity assay. NSCs were isolated from different ages and regions from mouse brain. A self-renewal NSCs population was obtained by culturing NSCs in DMEM/F12 with N2 supplement and bFGF (20 ng/ml) in coated Poly-l-lysine dish.

To quantify the percentage of alive vs. dead cells from the retrovirus infection, we counted the number of necrotic (GFP-) per 0.1 mm area of individual coverslip compared to the number of GFP + live cells, captured with high magnification differential interface contrast (DIC) and fluorescent images, that allows the simultaneous assessment of the GFP signal and necrotic features of NSCs.

#### *In vivo* analysis of SVZ stem cells proliferation and quantification in *Stat1*-/- and WT mice

Mice were injected ip (intraperitoneally) with BrdU (5-bromo-2′-deoxyuridine, 120 mg/kg body weight, Sigma Aldrich) once a day for seven consecutive days and were killed 2 h. after the last injection as previously described (Pluchino et al., 2008; Rasmussen et al., 2011).

### Immunohistochemistry *in vivo* and *in vitro* and quantification

For confocal imaging animals are perfused intracardially with 10 ml of 4% paraformaldehyde in PBS. The brain and spinal cord are removed and embedded in O.C.T., quick frozen in liquid nitrogen and kept at −70°C until sectioning. Cryostat sections (10 μm) of spinal cords are fixed with acetone or 4% paraformaldehyde and then labeled with the antibody of interest, as previously described (Imitola et al., 2003, 2004b; Aharoni et al., 2005; Arien-Zakay et al., 2009). Brains were placed in 30% sucrose for at least 24 h for cryoprotection. Coronal blocks of brain tissue from bregma –2 to + 2 mm were frozen in cryo-protective O.C.T.-solution (Sakura Finetek, Torrance, CA) at –80°C. The tissue was cut into floating sections of 40 μm thickness on a freezing microtome. Floating sections were incubated in a 2% solution of sodium borohydride for 20 min to reduce autofluorescence and then denatured by incubation in 2M hydrochloric acid for 2 h. at room temperature before staining. Sections were blocked with 8% horse serum for 1 h. and incubated overnight with rat antibody against BrdU (1:250, Accurate Chemical, Westbury, NY) and neural antibodies. For differentiation markers we incubated with primary antibodies overnight and secondary antibodies for 2 h in blocking solution. We use highly cross-adsorbed secondary antibodies to avoid cross-reactivity (Alexa 488 and Alexa 594). Confocal microscopy is performed using a Zeiss Laser Scanning Microscope 3D analysis software (Zeiss, Thornwood, NY) with a multitrack acquisition protocol to avoid potential overlapping of the two fluorochromes, all antibodies used in the study are shown in Table 1.

**TABLE 1:** Antibodies name and catalog numbers used in the study.

Western blot		CAT#	Company
Akt	Rabbit	4685	Cell Signaling Technology
P-Akt (S473)	Rabbit	4060	Cell Signaling Technology
β-actin	Mouse	SC-47778	Santa Cruz
β-catenin	Rabbit	8480	Cell Signaling Technology
GBK-3β	Rabbit	12456	Cell Signaling Technology
p-GBK-3β (S9)	Rabbit	5558	Cell Signaling Technology
HIF1A	Rabbit	Ab2185	Abcam
SoX9	Rabbit	ab26414	Abcam
STAT1 (Y701)	Rabbit	9167	Cell Signaling Technology
STAT3	Mouse	9139	Cell Signaling Technology
p-STAT3 (S727)	Rabbit	9134	Cell Signaling Technology
IHC		CAT#	Company
BMI-1	Rabbit	6964	Cell Signaling Technology
B_rd_U	Rat	OBT0030A	Accurate Chemical
DCX	Rabbit	4604	Cell Signaling Technology
GFAP	Mouse	3670	Cell Signaling Technology
IFN-γRI	Rabbit	MABF753	MilliporeSIGMA
MAP2	Rabbit	4542	Cell Signaling Technology
NESTIN	Mouse	33475	Cell Signaling Technology
O4	Mouse	MAB1326	R&D systems
SoX2	Goat	AF2018	R&D systems
STAT1	Rabbit	14994	Cell Signaling Technology
STAT1 (S727)	Rabbit	9177	Cell Signaling Technology
Tapasin1 (TAPBP)	Rabbit	66382	Cell Signaling Technology
Tenascin	Rabbit	33352	Cell Signaling Technology
FACS		CAT#	Company
CD4 Pacific Blue	RM4-5	558107	BD Bioscience
IFN-γPE-Cy7	XMG1.2	561040	BD Bioscience
IL-6 PE	MP5-20F3	551473	BD Bioscience
IL-10 APC	JES5-16E3	554707	BD Bioscience
TNF-α FITC	MP6-XT22	561064	BD Bioscience

The number of BrdU + cells in the corpus callosum was quantified from a 20 μm thick z-stack from 200 × 200 μm square right below the genu of the corpus callosum, while the number of BrdU + cells and the labeling index for Sox2 in the lateral wall were quantified from a 20 μm thick z-stack over 200 μm extending down from the corner of the SVZ. Cells labeled with Sox2 and/or BrdU were quantified using a using a 63 × water-immersion objective lens. The number of cells was averaged from four coronal sections per animal 120 μm apart starting at rostrocaudal level + 1.10 mm from bregma with four mice (Paxinos and Franklin, 2001) per experimental group.

*In vitro* NSCs were cultures as described above in PLL coated coverslip and at the end of the experimental setting PFA 4% was used for fixation. We performed stereological techniques to quantify neurospheres and differentiation with specific antibodies including MAP2, DCX, GFAP, and O4. Differentiated cells were counted using stereological technique using 10 areas per well, the total number of differentiated cells per antibody were counted per each well and expressed as total numbers or percentage of the total number of cells, that demonstrate switch in differentiation of typical NSCs cultures *in vitro* that are characterized by more astrocytic than neuronal differentiation.

### Confocal analysis, reconstruction, and pixel intensity analysis

Regions of interest around the lateral ventricle were analyzed with a confocal microscope (LSM 510 Laser Scanning Microscope and LSM 3D analysis software, Linux, Ogdensburg, NY). We use pixel intensity quantitative measurements to colocalize the staining of two molecules in 3D as we have shown previously (Imitola et al., 2004a,b; Wang et al., 2008; Rasmussen et al., 2011; Starossom et al., 2019).

#### Collection and flow cytometry staining of CSF samples from EAE mice and controls

Mice were anesthetized with ketamine (100 mg/kg body weight) and xylazine (10 mg/kg body weight). An incision was made in the neck and the membrane covering the cisterna magna was exposed. The membrane was perforated with an ultra-fine 29G insulin syringe and 2–10 μl CSF aspirated as previously described (Kivisäkk et al., 2009). Macroscopically blood contaminated samples were discarded.

CSF cells were collected from animals that had a clinical score > 2 and were washed once with PBS containing 5% serum for intracellular cytokine staining before being stimulated in full culture medium (RPMI supplemented with 10% FCS, glutamine, 2-ME, sodium pyruvate non-essential amino acids, HEPES and antibiotics; all from BioWhittaker) containing phorbol 12-myristate 13-acetate (PMA, 50 ng/ml, Sigma), ionomycin (1 μg/ml, Sigma), and monensin (GolgiStop, 0.65 μl/ml, BD Biosciences) for 4 h at 37°C. After staining of surface markers (CD4, Vβ3.2, Vβ11, CD69, and CD25), cells were fixed and permeabilized using Cytofix/Cytoperm (BD Biosciences) according to the manufacturer’s instructions. Cells were incubated with anti-cytokine antibodies (IFN-γ and IL-17) at RT for 20 min and washed twice in Perm/Wash buffer (BD Biosciences) before analysis. The following mAbs were used: CD4 Pacific Blue (clone RM4-5), IFN-γ PE-Cy7 (XMG1.2), IL-10 APC (JES5-16E3), IL-6 PE (MP5-20F3) and TNF-a FITC (MP6-XT22) from BD Biosciences. Samples were acquired on a FACS Calibur or an LSR II flow cytometer (BD Biosciences) and analyzed using FlowJo version 8.4.3 (Tree Star, Inc.).

### RNA Isolation

RNA was isolated from the cells using the RNeasy Mini Qit (Qiagen, Valencia, CA) and consistently yielded high quality RNA based on post analysis of multiple parameters including visualization of the 28S and 18S bands on a 1% agarose RNA gel, spectrophotometric readings of 1.8–2.0 as determined by [(Abs 260–Abs 320)/(Abs 280–Abs 320)], and visualization of two distinct peaks on a bioanalyzer (Agilent Technologies, Andover MA, USA) representing the 28S and 18S ribosomal RNA.

### Protein extraction and western blot analysis

Cells were harvested at different time points and washed with ice-cold PBS. Whole cell lysates were prepared by extraction in lysis buffer containing 1% Triton X-100, 20 mM Tris-HCl pH 8.0, 150 mM NaCl, 3 mM sodium pyrophosphate, 10% glycerol, 2 mM sodium orthovanadate, and 20 μl/ml Protease Arrest (Genotech, St. Louis, MO, USA). Protein concentration was determined by the Bradford protein assay (Bio-Rad, Hercules, CA, USA). 40 μg of protein was analyzed by SDS-PAGE using 10% Tris-glycine gels (Invitrogen, Carlsbad, CA, USA), followed by transfer to Hybond-P membranes (Amersham, Piscataway, NJ, USA). Membranes were blocked in 5% non-fat milk in TBS containing 0.05% Tween 20 for 1 h and incubated with primary antibodies at 4°C overnight. The detection step was performed with HRP-coupled anti-rabbit IgG antibodies (Genotech). Blots were developed with the ECL plus chemiluminescence detection system (Amersham, Piscataway, NJ, USA).

We extracted proteins from NSCs and from brain tissues of adult mice in lysis buffer [1 times phosphate-buffered saline, 5 mM EDTA, 1% Triton X-100 and protease inhibitor cocktail tablet (Roche)]. We will separate immunoprecipitated proteins by SDS-PAGE, immunoblot them and incubate them with the appropriate primary antibodies overnight at 4°C and then with the corresponding horseradish peroxidase–conjugated secondary antibodies for 1–2 h at room temperature. Proteins will be detected using ECL chemiluminescence (Amersham).

### Microarray and interferon stimulated gene single cell RNAseq analysis

For gene expression analysis mRNA from duplicates NSCs exposed to interferon-γ for 24 h, were processed on Affymetrix microarrays and genes with change expression values ≥ 2-fold and statistical significance by *t*-test with a Bonferroni correction, were confirmed using a different microarray platform, confocal microscopy, and western blot. Before determining differential expression between *Stat1*^+/+^ and *Stat1*^–/–^ NSCs, genes showing less than 0.25 variance were first filtered out using the varFilter function in the genefilter R package. Next, differential expression was performed using the limma R package, comparing the knockout to control, and using to FDR to correct for multiple hypotheses (Ritchie et al., 2015). A heatmap of DEGs was then generated using the online matrix visualization software, Morpheus.^2^ To visualize gene ontology enrichment, the ClueGO plug-in in Cytoscape was utilized, using standard parameters, except for including GO Fusion (Bindea et al., 2009). PPI networks from all DEGs at FDR < 0.15 were produced using StringDB (Szklarczyk et al., 2019). Finally, *in situ* hybridization images of sagittal sections of embryonic mouse brains for selected DEGs were retrieved from Brainspan (Lein et al., 2007).

Normalized TPM values and cell annotation data for the 10× single cell RNA-seq dataset from were retrieved directly from https://github.com/Martin-Villalba-lab/Data/tree/master/Cell_2019 (Kalamakis et al., 2019). Original cell annotations were preserved and transferred to a new SeuratObject in which all genes were examined using the Seurat package in R (Hao et al., 2021). *Stat1* target genes were defined using a comprehensive list from Chip-Seq analysis (Satoh and Tabunoki, 2013). Direct target genes were defined by those at which *Stat1* bound to promoters, since intronic localization could be *Stat1* binding enhancers of unknown far-away target genes, and genes that had at least two log-fold change increase by treatment with *Stat1* activator, IFN-γ.

#### Real-time PCR of NSCs

Total RNA is extracted from NSCs using RNeasy mini kit (Qiagen, Valencia, CA, USA), and cDNA is synthesized using SuperScript™ III first-strand synthesis system for RT-PCR (Invitrogen, Carlsbad, CA, USA). Expression of *Stat1* (as an example of a gene of interest) and glyceraldehyde phosphate dehydrogenase (GAPDH) or Hypoxanthine Guanine Phosphoribosyltransferase (HPRT) mRNA, reliable housekeeping genes for NSCs studies, (Chapman and Waldenstrom, 2015; Kang et al., 2019) were measured by PCR in separate tubes in duplicates using probes labeled with 6-carboxyfluorescein (FAM) and VIC^®^, respectively, TAMRA^®^ as a quencher, TaqMan^®^ universal PCR master mix and the ABI Prism 7700 sequence detection system (all from Applied Biosystems, Foster City, CA, USA). Primers and probe for GAPDH were purchased from Applied Biosystems. A comparative threshold cycle (C_T_) will be used to determine mRNA expression of Stat1 and GAPDH relative to no-template control (calibrator). C_T_ value will be normalized for each sample using the formula: ΔC_T_ = C_T (stat1)_- C_T (GAPDH)_ and the relative expression of stat1 will be then calculated using the expression 2^–Δ^ ^CT^.

#### ChIP and qPCR and luciferase assay

Neural stem cells were isolated from *in vitro* cultures in the presence or absence of IFN-γ purified and ChIP was performed with ChampionChip kit (SABiosciences). Cell lysates were used for immunoprecipitation with anti-Stat1 (Abcam) and anti-Sox9 (Abcam) and were compared to control IgG. Several regions of the Sox9 promoter containing putative *Stat1* binding sites were amplified by SYBR Green qPCR (Roche) and quantified in triplicate with the percent of input method. The following primers were used for *Sox9:* site 1F: 5′-CACACACACACACACACACACA*-3*′, site 1R: 5′-GGGACGTCCGAGTGTGTAA*-3*′; site 2F: 5′-AGAGCGCTGCTCGGAACT*-3*′, site 2R: 5′-CCACTTGCACCTCGTCTCTC*-3*′; and site 3F: 5′-GCAGCTGAGGGAAGAGGAG*-3*′, site 3R: 5′-GAAGGGGTCCAGGAGATTCA*-3*′. qPCR quantification of ChIP assays on NSCs from adult mice treated with or without IFN-γ for 4 h. Samples were immunoprecipitated with an anti-Stat1 antibody and amplified with *Sox9* or irrelevant control primers. For negative controls, samples were processed without IgG antibody. Vectors for *Sox9* promoters (Kumar and Lassar, 2009) were used for transcriptional luciferase assay. Multiple Reporter vectors coding for the Firefly Luciferase under the control of the Sox9 promoter encompassing nucleotides −6,028 to +300 bp, reporter assays were carried out as described previously (Elyaman et al., 2012). In brief, 293T cells were transfected with 0.4 μg of the reporter vector coding for the firefly luciferase (pGL3 basic; Promega) under the control of the Sox9 promoter and with 0.8 μg of the *Stat1*. Cells were cultured for 48 h. before harvesting and the relative Sox9 promoter activity was measured with Promega kit in accordance with the manufacturer’s instructions.

### Statistical analysis

All data is presented as mean ± SEM, statistical analysis was performed using the unpaired, two-sided *t*-test comparison between EAE and control or by using one-way analysis of variance (ANOVA) with Turkey’s Multiple Comparison Test. Significant differences were assumed at the 5% level and represented as *P*-values (*p* < 0.05).

## Data availability statement

The original contributions presented in the study are included in the article/Supplementary material, further inquiries can be directed to the corresponding author/s upon reasonable request.

## Ethics statement

The animal study was reviewed and approved by the Animal Care Committee of Harvard University.

## Author contributions

JI and SK conceived the project. JI, EH, FW, MO, WE, SS, and PK performed the experiments. JI, EH, and SK wrote the manuscript with contributions from all authors. All authors read and approved the final manuscript.

## Footnotes

1
http://developingmouse.brain-map.org/

2
https://software.broadinstitute.org/morpheus/

## Supplementary material

Supplementary Figure 1 Quantification of cell death after STAT1 infection, Validation of SVZ NSCs by qPCR, genotype and Retrovirus targeting of *Stat1*^–/–^ NSCs. Quantification of cell death after STAT1 infection: **(A)** cell death quantification of GFP cells after MSCV and STAT1 vectors, there is a minimal but significant increase in the cell death of STAT1 vector expressing cells. Validation of the absence of *Stat1* in SVZ NSCs by qPCR, genotype: **(B)** diagram of the SVZ microdissected area used to perform isolation of NSCs from brains of *Stat1*^–/–^ and mice and validation of *Stat1*^–/–^ genotypes used for the SVZ NSCs isolation, the gel represents typical genomic DNA confirmation in mice tissue and cultures neurospheres, this is done to verify the fidelity of the NSCs cultures from *Stat1*^–/–^ and controls. **(C)** qPCR analysis of neurospheres to confirm reduction of expression Stat1 in NSCs from *Stat1*^–/–^ deficient mice. **(D)** Validation of effects of *Stat1* retrovirus in *Stat1* Neurospheres from *Stat1*^–/–^ NSCs: NSCs were dissociated and infected with retrovirus delivering STAT1-GFP, the cells were sorted by FACS 48 hours after infection based on GFP expression and plated at clonal dilution in FGF-2 medium. **(E)** Representative image showing the reduction of the numbers (*p* < 0.001 by *t*-test) and **(F)** size of secondary neurospheres in the *Stat1*^–/–^ NSCs treated with the STAT1-GFP retrovirus compared to murine stem cell virus (MSCV)-GFP vector after 7 days in cultures (*p* = 0.019).

Supplementary Figure 2 Differentiation of *Stat1*^–/–^ NSCs compared to *Stat1*^ + ⁣/ +^. **(A)** Staining of doublecortin DCX+ cells (green) and GFAP+ cells (red) in WT (left panel) and STAT1-/- (right panel). **(B)** Quantification of DCX+ and GFAP+ cells in *Stat1*^ + ⁣/ +^ and *Stat1*^–/–^ in mm^3^ (*p* < 0.001 by *t*-test).

Supplementary Figure 3 Expression of interferon related genes in subventricular zone NSCs and IFN-γ T cells in EAE CSF. **(A)** Diagram and Electron Microscopy of the relationship of NSCs with the CSF, ciliated NSCs cells (Type B) protrude the cytoplasm in the CSF interacting with the cytokines from the CSF. **(B)** Comparative microarray gene expression of interferon-γ related genes in NSCs (*blue bars*) compared to normal brain (*red bars*), showing constitutive expression of these genes in isolated SVZ NSCs expression values are given in Affymetrix mean fluorescence. **(C)** Expression of IFN-γ receptor-1 (*green*) in the ventricular and SVZ mouse brain. NSCs are co-stained with the neural stem cell regulator Bmi-1 (*red*) bar = 20 μm. **(D)** Quantitative PCR analysis of *Stat1* expression of isolated SVZ from SJL mice with EAE, results from *n* = 3 animals per group. **(E)** Immune cells from draining lymph nodes, CSF, blood, and MEN of animals with EAE (day 14 post-immunization) were isolated by dissection of meninges and stained intracytoplasmically for IFN-g and IL-17 showing a higher percentage of IFN-γ producing CD4+ T cells in EAE in the PLP-relapsing remitting EAE compared to IL-17 (*p* < 0.001). *n* = 3 animals per group. **(F)** Confirmation in a different EAE model, using 2D2 mice, quantification of numbers of MOG-specific IFN-γ producing T cells in the CSF compared to different immune compartments. IFN-γ producing cells in CSF of diseased animals, 80% compared to 20% of IL-17 producing cells in CSF, data represent *n* = 3 animal per group.

Supplementary Figure 4 Effects of IFN-γ in long term cultures of NSCs inducing alteration of lifespan. **(A)** Quantification of the numbers of secondary neurospheres by frequency exposed to various doses of IFN-γ showing a significant dose dependent response *p* < 0.001 by *t*-test. **(B)** Western blot of IFN-γ induced phosphorylation of STAT1 at tyrosine Y705 but not STAT3 in serine S727 compared to total STAT-1 and STAT-3 (*bottom lane*). **(C)** Representative FACS showing no increase apoptosis or toxicity of IFN-γ used for experiments showing decrease proliferation after day 5 in culture. **(D,E)** Quantification of *tapasin-1* and *Tenascin* after IFN-γ treatment *in vitro* and **(F)** confirmation of proteins by confocal microscopy. **(H)** Quantification of NSCs lifespan after repeated passages of primary isolated (*p5*) compared to late passage (*p60*) NSCs in the presence of IFN-γ. The red line below the *X*-axis indicates the presence of IFN-γ in the culture. The blue lines are cells passaged p5 times while the red lines are the cells passaged p60 times, the dashed lines are cells cultured in the presence of IFN-γ while the continuous lines are cells not exposed to IFN-γ **(I)** example of neurospheres growing in the absence or presence of long term IFN-γ 100 U/ml, showing a significant decrease in the size of neurospheres and single NSCs that failed to form neurospheres but still viable (*insert*).
