# Supplementary figures and images for: Stat1 is an inducible transcriptional repressor of neural stem cells self-renewal program during neuroinflammation
Source: Front Cell Neurosci. 2023 Aug 16;17:1156802. doi: 10.3389/fncel.2023.1156802 (PMC10469489; doi:10.3389/fncel.2023.1156802)

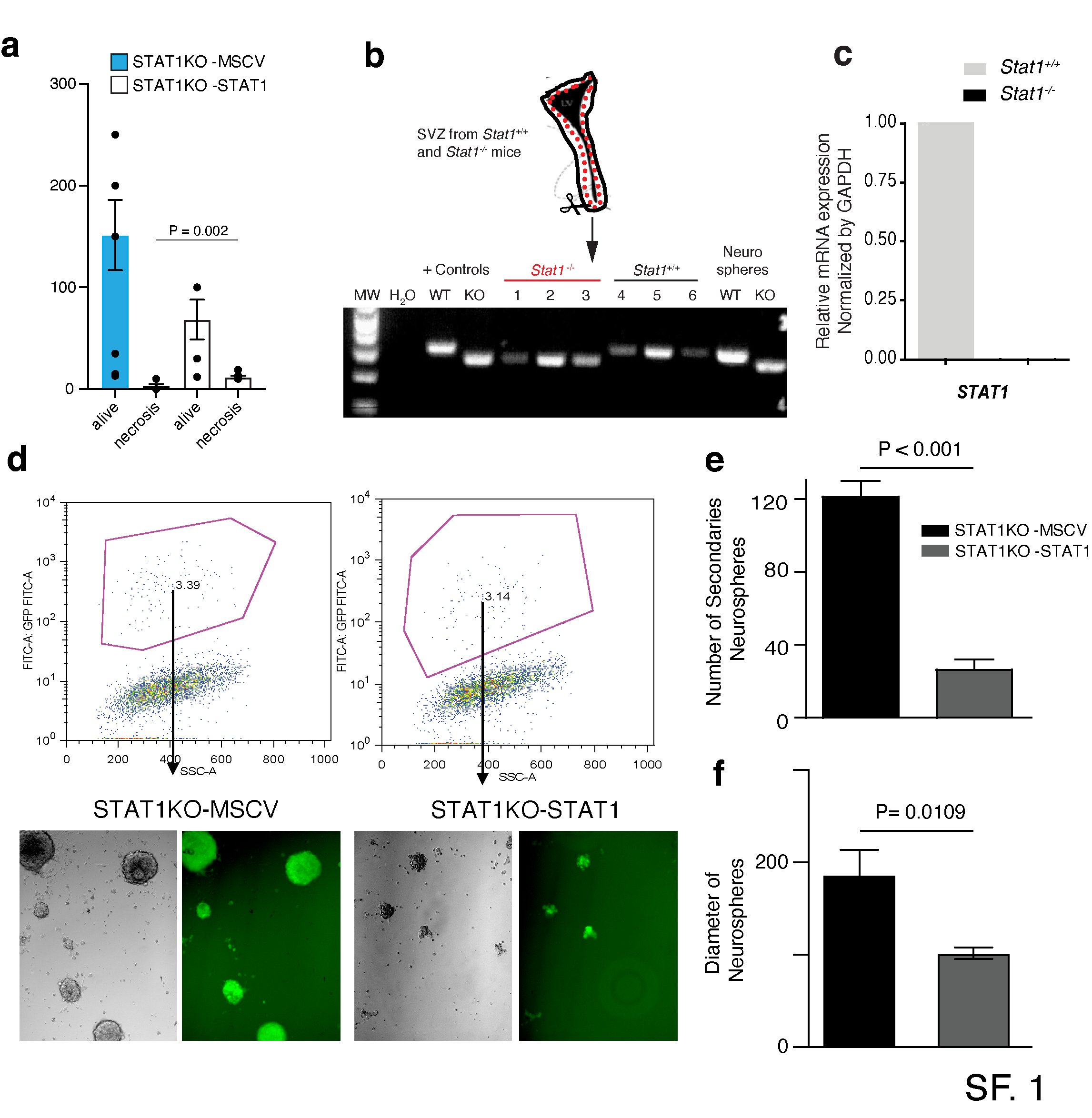

Supplement: Supplementary Figure 1 — Quantification of cell death after STAT1 infection, Validation of SVZ NSCs by qPCR, genotype and Retrovirus targeting of Stat1–/– NSCs. Quantification of cell death after STAT1 infection: (A) cell death quantification of GFP cells after MSCV and STAT1 vectors, there is a minimal but significant increase in the cell death of STAT1 vector expressing cells. Validation of the absence of Stat1 in SVZ NSCs by qPCR, genotype: (B) diagram of the SVZ microdissected area used to perform isolation of NSCs from brains of Stat1–/– and mice and validation of Stat1–/– genotypes used for the SVZ NSCs isolation, the gel represents typical genomic DNA confirmation in mice tissue and cultures neurospheres, this is done to verify the fidelity of the NSCs cultures from Stat1–/– and controls. (C) qPCR analysis of neurospheres to confirm reduction of expression Stat1 in NSCs from Stat1–/– deficient mice. (D) Validation of effects of Stat1 retrovirus in Stat1 Neurospheres from Stat1–/– NSCs: NSCs were dissociated and infected with retrovirus delivering STAT1-GFP, the cells were sorted by FACS 48 hours after infection based on GFP expression and plated at clonal dilution in FGF-2 medium. (E) Representative image showing the reduction of the numbers (p < 0.001 by t-test) and (F) size of secondary neurospheres in the Stat1–/– NSCs treated with the STAT1-GFP retrovirus compared to murine stem cell virus (MSCV)-GFP vector after 7 days in cultures (p = 0.019). [file Image_1.TIF]

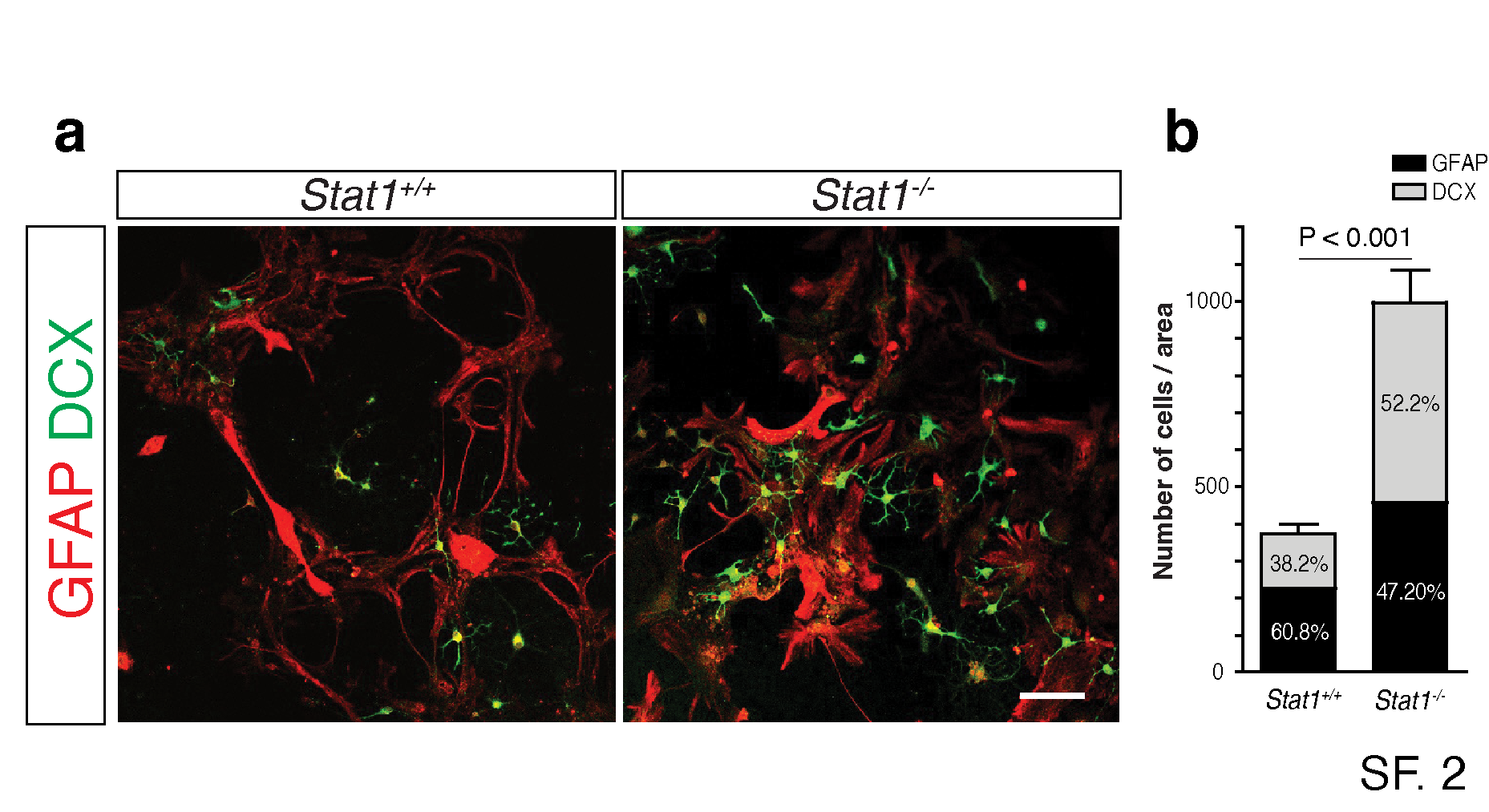

Supplement: Supplementary Figure 2 — Differentiation of Stat1–/– NSCs compared to Stat1 + ⁣/ +. (A) Staining of doublecortin DCX+ cells (green) and GFAP+ cells (red) in WT (left panel) and STAT1-/- (right panel). (B) Quantification of DCX+ and GFAP+ cells in Stat1 + ⁣/ + and Stat1–/– in mm3 (p < 0.001 by t-test). [file Image_2.TIF]

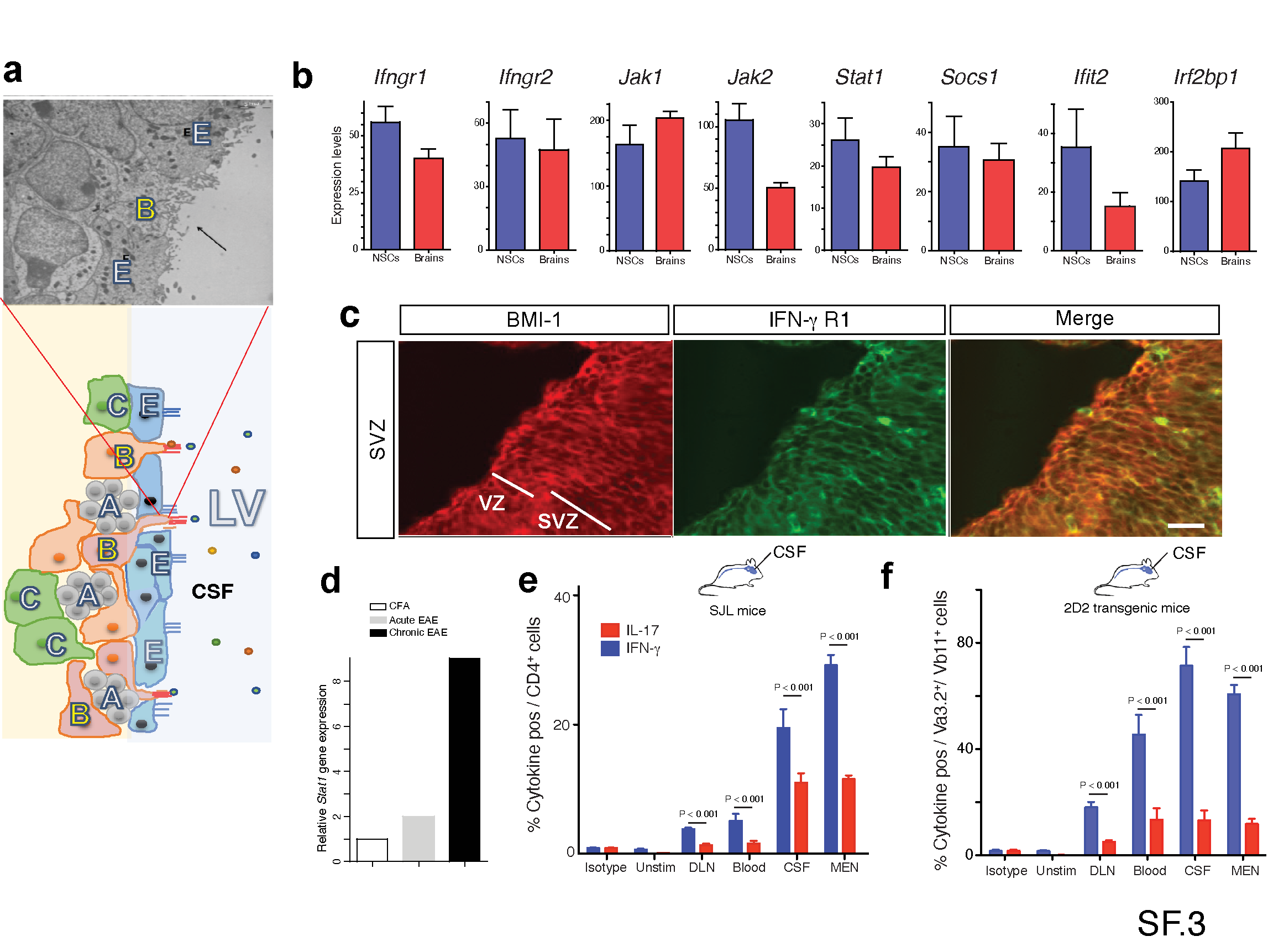

Supplement: Supplementary Figure 3 — Expression of interferon related genes in subventricular zone NSCs and IFN-γ T cells in EAE CSF. (A) Diagram and Electron Microscopy of the relationship of NSCs with the CSF, ciliated NSCs cells (Type B) protrude the cytoplasm in the CSF interacting with the cytokines from the CSF. (B) Comparative microarray gene expression of interferon-γ related genes in NSCs (blue bars) compared to normal brain (red bars), showing constitutive expression of these genes in isolated SVZ NSCs expression values are given in Affymetrix mean fluorescence. (C) Expression of IFN-γ receptor-1 (green) in the ventricular and SVZ mouse brain. NSCs are co-stained with the neural stem cell regulator Bmi-1 (red) bar = 20 μm. (D) Quantitative PCR analysis of Stat1 expression of isolated SVZ from SJL mice with EAE, results from n = 3 animals per group. (E) Immune cells from draining lymph nodes, CSF, blood, and MEN of animals with EAE (day 14 post-immunization) were isolated by dissection of meninges and stained intracytoplasmically for IFN-g and IL-17 showing a higher percentage of IFN-γ producing CD4+ T cells in EAE in the PLP-relapsing remitting EAE compared to IL-17 (p < 0.001). n = 3 animals per group. (F) Confirmation in a different EAE model, using 2D2 mice, quantification of numbers of MOG-specific IFN-γ producing T cells in the CSF compared to different immune compartments. IFN-γ producing cells in CSF of diseased animals, 80% compared to 20% of IL-17 producing cells in CSF, data represent n = 3 animal per group. [file Image_3.TIF]

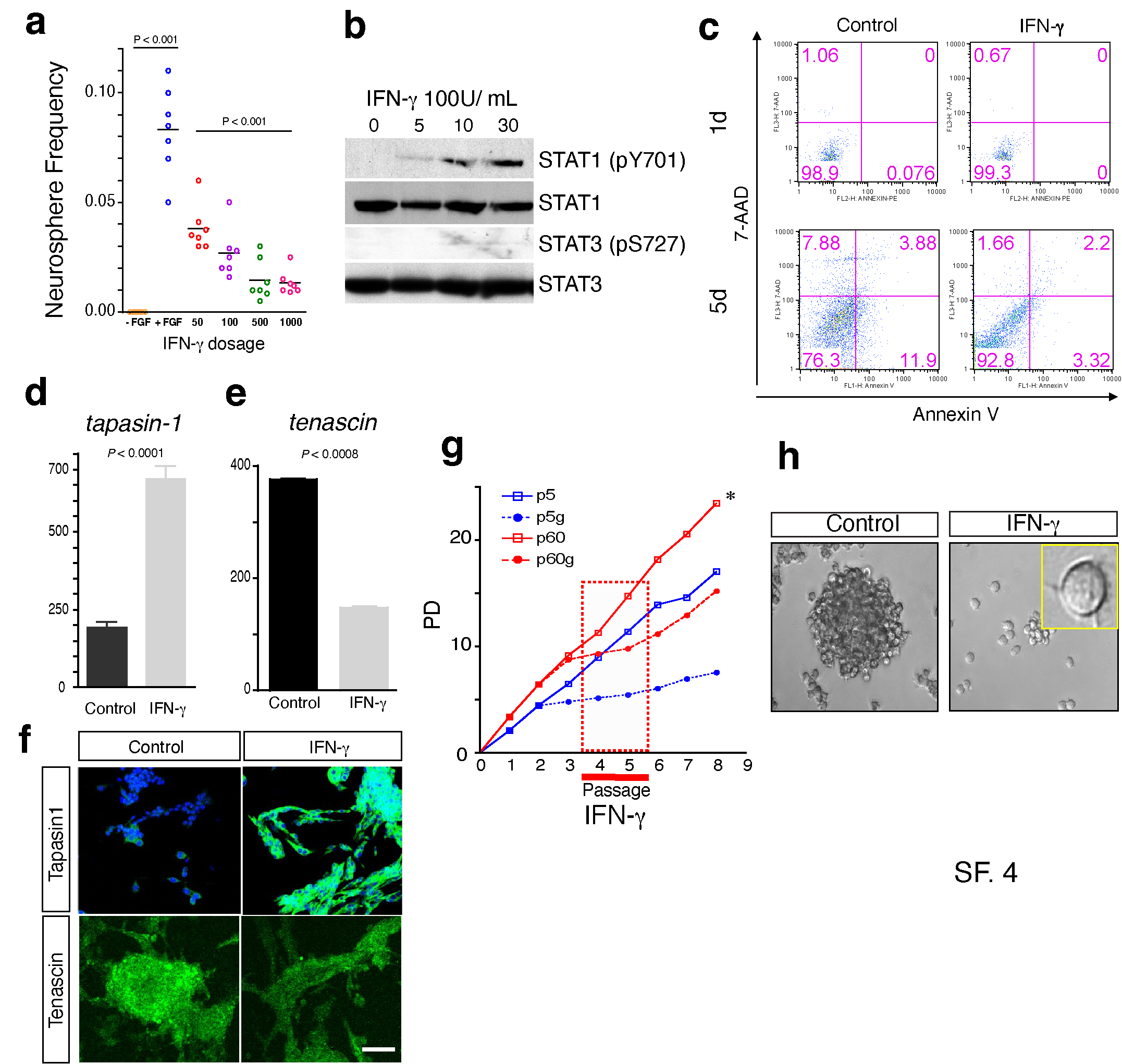

Supplement: Supplementary Figure 4 — Effects of IFN-γ in long term cultures of NSCs inducing alteration of lifespan. (A) Quantification of the numbers of secondary neurospheres by frequency exposed to various doses of IFN-γ showing a significant dose dependent response p < 0.001 by t-test. (B) Western blot of IFN-γ induced phosphorylation of STAT1 at tyrosine Y705 but not STAT3 in serine S727 compared to total STAT-1 and STAT-3 (bottom lane). (C) Representative FACS showing no increase apoptosis or toxicity of IFN-γ used for experiments showing decrease proliferation after day 5 in culture. (D,E) Quantification of tapasin-1 and Tenascin after IFN-γ treatment in vitro and (F) confirmation of proteins by confocal microscopy. (H) Quantification of NSCs lifespan after repeated passages of primary isolated (p5) compared to late passage (p60) NSCs in the presence of IFN-γ. The red line below the X-axis indicates the presence of IFN-γ in the culture. The blue lines are cells passaged p5 times while the red lines are the cells passaged p60 times, the dashed lines are cells cultured in the presence of IFN-γ while the continuous lines are cells not exposed to IFN-γ (I) example of neurospheres growing in the absence or presence of long term IFN-γ 100 U/ml, showing a significant decrease in the size of neurospheres and single NSCs that failed to form neurospheres but still viable (insert). [file Image_4.TIF]
